# Supplementary material for: Hearing Impairment Overview in Africa: the Case of Cameroon
Source: Genes (Basel). 2020 Feb 22;11(2):233. doi: 10.3390/genes11020233 (PMC7073999; doi:10.3390/genes11020233)
Supplement: Supplementary file 1 [file genes-11-00233-s001.pdf]

SUPPLEMENTARY MATERIALS

Hearing Impairment Overview in Africa: The Case of Cameroon

Edmond Wonkam Tingang, Jean Jacques Noubiap, Jean Valentin F. Fokouo, Oluwafemi Gabriel Oluwole, Séraphin Nguefack, Emile R. Chimusa and Ambroise Wonkam

Content

Table S1: Search strategy in Pubmed 2
Table S2: Search strategy in Scopus 2
Table S3: Search strategy in AFROLIB 2
Table S4: Search strategy in African Index Medicus 2
Table S5: Search strategy in African Journals Online 3
Table S6: Age at diagnosis of hearing impairment in Cameroon (extracted from the study by Wonkam et al. [1]) 3
Table S7: Categories of hearing loss in Cameroon with pure tone audiometry, according to the BIAP classification (extracted from the study by Wonkam et al. [1])........ 3
Figure S1: Inheritance of familial hearing impairment in Cameroon. (A) Pedigree of a consanguineous family with autosomal recessive non-syndromic hearing impairment. (B) Pedigree of a family with non-syndromic hearing impairment suggestive of mitochondrial inheritance. Arrows here indicate the probands (Extracted from the study by Tingang Wonkam et al. [2]). 4

Table S1: Search strategy in Pubmed

Search step	Search terms	Hits
**1**	"Hearing impairment" OR "hearing loss" OR deaf OR deafness	99 075
**2**	Cameroon	
**3**	#1 and #2	31
**4**	#3 and **Search limits:** from inception to October 31th 2019	31

Table S2: Search strategy in Scopus

Search step	Search terms	Hits
**1**	"Hearing impairment" OR "hearing loss" OR deaf OR deafness	146 211
**2**	Cameroon	
**3**	#1 and #2	38
**4**	#3 and **Search limits:** publication year < 2020	38

Table S3: Search strategy in AFROLIB

Search step	Search terms	Hits
**1**	"Hearing impairment" "hearing loss" "deaf" "deafness" "Cameroon"	15
**2**	#1 and **Search limits:** publication date ≤ October 31th 2019	8

Table S4: Search strategy in African Index Medicus

Search step	Search terms	Hits
**1**	"Hearing impairment" "hearing loss" "deaf" "deafness" "Cameroon"	83
**2**	#1 and **Search limits:** publication date ≤ October 31th 2019	81

Table S5: Search strategy in African Journals Online

Search step	Search terms	Hits
**1**	“Hearing impairment” OR “hearing loss” OR deaf OR deafness	268
**2**	#1 and **Search limits:** from inception to October 31th 2019	175

Table S6: Age at diagnosis of hearing impairment in Cameroon (extracted from the study by Wonkam et al. [1])

**Age of onset**		**Number of cases, n (%)**
Prelingual	(Before 2 years old)	437 (75.1)
Perilingual	(Between 2 and 4 years)	116 (20)
Postlingual	(After 4 years)	29 (4.9)

Table S7: Categories of hearing loss in Cameroon with pure tone audiometry, according to the BIAP classification (extracted from the study by Wonkam et al. [1]).

**Category of hearing loss**	**Sensorineural hearing loss**	**Mixed hearing loss**	**Total, n (%)**
Severe I (71–80 dB)	14	11	25 (4.8)
Severe II (81–90 dB)	39	14	53 (10.1)
Profound I (91–100 dB)	140	27	167 (31.9)
Profound II (101–110 dB)	156	16	172 (32.8)
Profound III (111–120 dB)	71	11	82 (15.6)
Total (>120 dB)	25	0	25 (4.8)
Total, n (%)	445 (84.9)	79 (15.1)	524 (100)

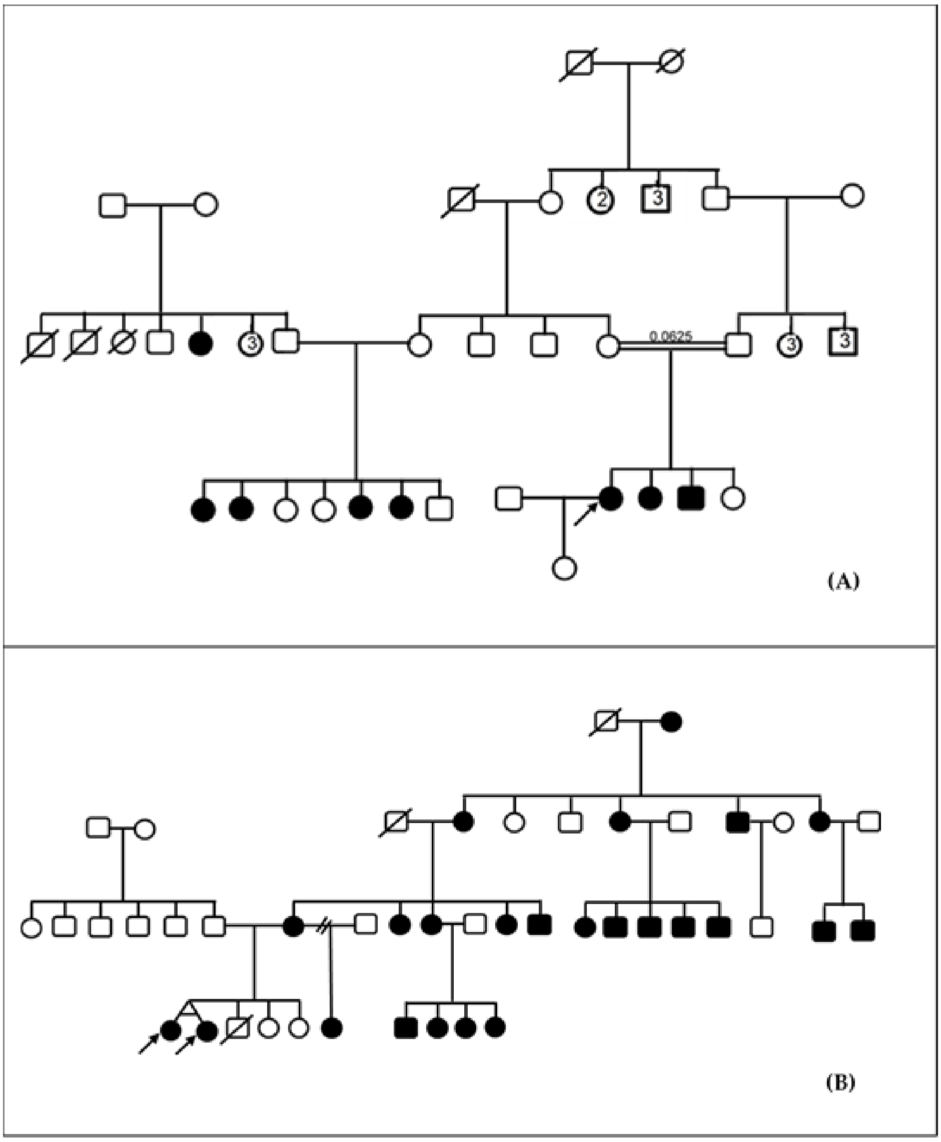

Figure S1: Inheritance of familial hearing impairment in Cameroon. (A) Pedigree of a consanguineous family with autosomal recessive non-syndromic hearing impairment. (B) Pedigree of a family with non-syndromic hearing impairment suggestive of mitochondrial inheritance. Arrows here indicate the probands (Extracted from the study by Tingang Wonkam et al. [2]).

Reference:

1. Wonkam, A.; Noubiap, J.J.N.; Djomou, F.; Fieggen, K.; Njock, R.; Toure, G.B. Aetiology of childhood hearing loss in Cameroon (sub-Saharan Africa). *Eur J Med Genet* **2013**, *56*, 20–25.
2. Tingang Wonkam, E.; Chimusa, E.; Noubiap, J.J.; Adadey, S.M.; F Fokouo, J.V.; Wonkam, A. GJB2 and GJB6 Mutations in Hereditary Recessive Non-Syndromic Hearing Impairment in Cameroon. *Genes (Basel)* **2019**, *10*.
